# Circulating fibroblast growth factor-2 precipitates HIV nephropathy in mice

**DOI:** 10.1242/dmm.048980

**Published:** 2021-07-26

**Authors:** Jharna R. Das, Marina Jerebtsova, Pingtao Tang, Jinliang Li, Jing Yu, Patricio E. Ray

**Affiliations:** 1 Children's National Hospital, Washington, DC 20010, USA; 2 Department of Pediatrics, The George Washington University School of Medicine, Washington, DC 20052, USA; 3 Child Health Research Center, Department of Pediatrics, University of Virginia School of Medicine, Charlottesville, VA 22908, USA

**Keywords:** HIV nephropathy, Fibroblast growth factor-2, Children, HIV kidney diseases

## Abstract

People of African ancestry living with the human immunodeficiency virus-1 (HIV-1) are at risk of developing HIV-associated nephropathy (HIVAN). Children with HIVAN frequently show high plasma fibroblast growth factor-2 (FGF-2) levels; however, the role of circulating FGF-2 in the pathogenesis of childhood HIVAN is unclear. Here, we explored how circulating FGF-2 affected the outcome of HIVAN in young HIV-Tg_26_ mice. Briefly, we demonstrated that FGF-2 was preferentially recruited in the kidneys of mice without pre-existing kidney disease, precipitating HIVAN by activating phosphorylated extracellular signal-regulated kinase (pERK) in renal epithelial cells, without inducing the expression of HIV-1 genes. Wild-type mice injected with recombinant adenoviral FGF-2 (rAd-FGF-2) vectors carrying a secreted form of human FGF-2 developed transient and reversible HIVAN-like lesions, including proteinuria and glomerular enlargement. HIV-Tg_26_ mice injected with rAd-FGF-2 vectors developed more-significant proliferative and pro-fibrotic inflammatory lesions, similar to those seen in childhood HIVAN. These lesions were partially reversed by treating mice with the FGF/VEGF receptor tyrosine kinase inhibitor PD173074. These findings suggest that high plasma FGF-2 levels may be an independent risk factor for precipitating HIVAN in young children.

## Abstract (precis)

**Editor's choice:** We developed a new mouse model of childhood HIV-associated nephropathy (HIVAN) and showed that circulating FGF-2 precipitated HIVAN in young HIV-Tg_26_ mice by inducing the pERK pathway.

## INTRODUCTION

Children of African descent living with human immunodeficiency virus-1 (HIV-1) are at high risk of developing HIV-associated nephropathy (HIVAN) if not treated continuously with modern antiretroviral therapy (ART) throughout their childhood (Beng et al., 2020). Childhood HIVAN is characterized by the presence of heavy proteinuria in association with the de-differentiation and proliferation of podocytes and tubular epithelial cells, leading to the collapse of glomerular capillaries and/or focal segmental glomerulosclerosis, mesangial expansion, enlarged glomeruli and formation of tubular microcysts that cause renal enlargement and chronic kidney failure (Strauss et al., 1989; Ray et al., 1998b).

The main pathological paradigm of HIVAN is that the expression of HIV transcripts in renal epithelial cells precipitates this disease (Bruggeman et al., 2000; Marras et al., 2002). In addition, because HIVAN is mainly seen in people of sub-Saharan African ancestry, the increased risk of these individuals to develop HIVAN has been attributed in great part to the apoliprotein-1 (APOL1) genetic variants G1 and G2 (Genovese et al., 2010; Kopp et al., 2011). Overall, these variants increase the lifetime risk of young adults who are not receiving appropriate ART to develop HIVAN by ∼50% (Kopp et al., 2017). Nonetheless, the mechanisms through which HIV genes interact with the APOL1 risk variants to precipitate HIVAN are not clearly understood. Furthermore, given the relative low prevalence of the APOL1 risk variants in people of African ancestry, many children not carrying these risk variants develop HIVAN as well (Purswani et al., 2016; Ekulu et al., 2019). Thus, other yet unknown factors associated with sub-Saharan African ancestry should contribute to precipitate HIVAN.

Previous studies identified high levels of fibroblast growth factor-2 (FGF-2) in the plasma and urine of children with HIVAN and other HIV chronic kidney diseases (HIV-CKDs) (Ray et al., 1999). The accumulation of FGF-2 in the kidneys has been associated with the progression of several experimental and human kidney diseases (Floege et al., 1995; Mazue et al., 1993; Kriz et al., 1995; Morita et al., 1994). In addition, the urinary levels of FGF-2 are considered a promising candidate biomarker to follow the outcome of children with HIVAN (Soler-Garcia et al., 2009). However, the role of circulating FGF-2 in the pathogenesis of childhood HIVAN is unknown. Based on these studies, we hypothesized that sustained high circulating levels of FGF-2 can contribute to precipitate HIVAN and HIV-CKDs in children. To test this hypothesis, we injected recombinant human (rh) FGF-2 or adenoviral vectors carrying a secreted form of rh-FGF-2 to young wild-type (WT) and HIV-transgenic_26_ (HIV-Tg_26_) mice without pre-existing renal disease, and assessed their renal outcome during their first weeks of life. More specifically, our studies were focused on determining whether high levels of circulating FGF-2 are able to induce some of the typical features of HIVAN in mice, and developing a new mouse model system of childhood HIVAN.

## RESULTS

### Recruitment of circulating FGF-2 in the kidneys of young WT and HIV-Tg_26_ with renal disease

**Fig. 1.**
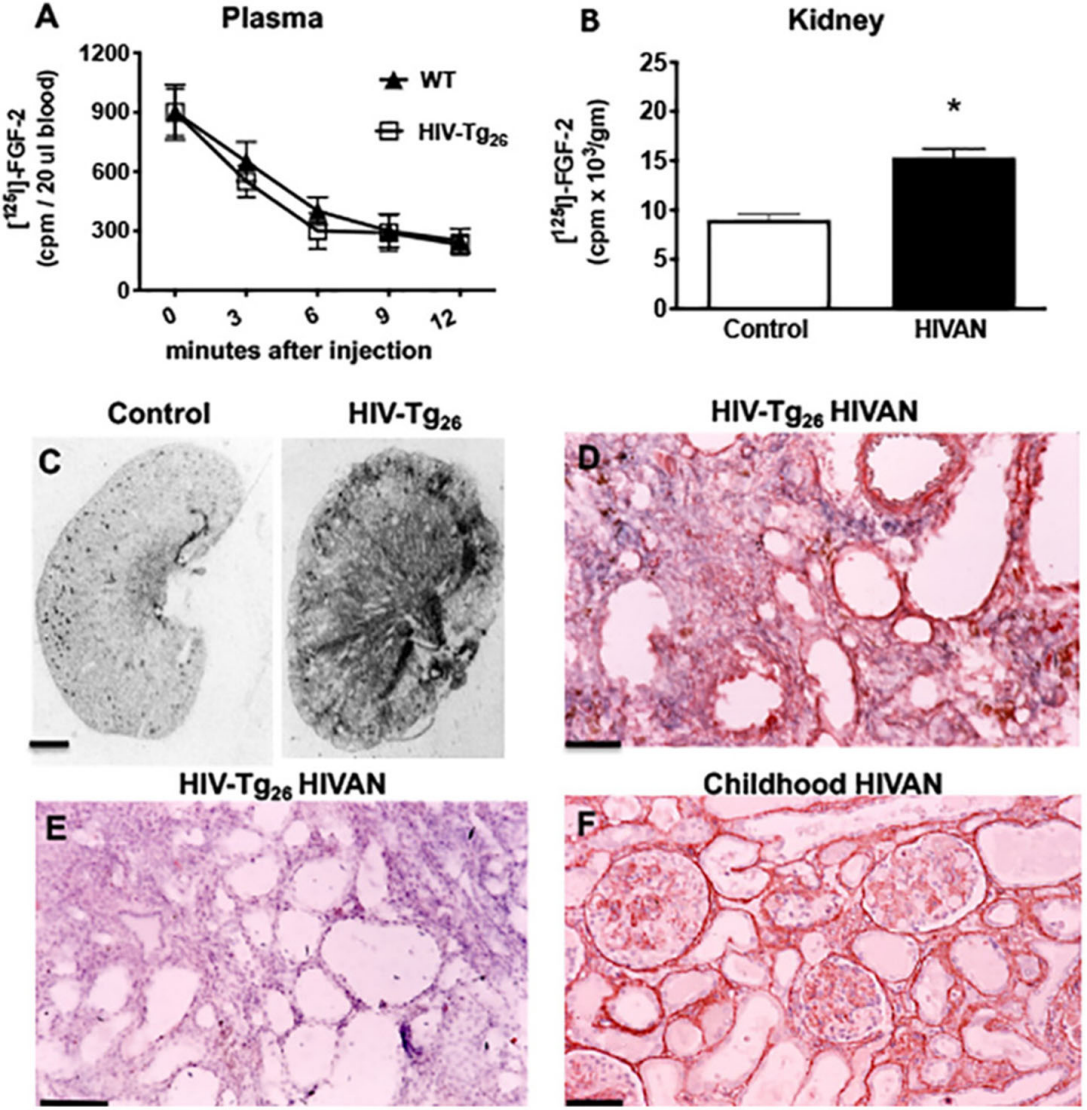
**Recruitment of circulating FGF-2 in the kidney of HIV-Tg_26_ mice with renal disease.** (A) Representative graph documenting the clearance of [^125^-I] FGF-2 from the blood of wild-type (WT) and HIV-Tg_26_ mice, 12 min after an intravenous FGF-2 injection. Bars represent the mean±s.e.m. [^125^-I] FGF-2 counts/min (cpm) values (*n*=3 mice per group). (B) Mean±s.e.m. for [^125^-I] FGF-2 accumulated in the kidney of WT and HIV-Tg_26_ mice with renal disease after an intravenous injection, as described in the Materials and Methods. *P<0.05 by the Mann–Whitney unpaired *t*-test (*n*=3 mice per group). (C) Representative autoradiographs showing the total [^125^-I]FGF-2 binding in the kidney of WT and HIV-Tg_26_ mice with renal disease (*n*=3 mice per group). (D) FGF-2 immunohistochemistry staining (red) in a representative renal section harvested from HIV-Tg_26_ mice with renal disease (*n*=4 samples). (E) Similar renal section harvested from an HIV-Tg_26_ mouse with renal disease incubated with a control non-specific IgG antibody as described in the Materials and Methods (*n*=3 samples). (F) Representative immunohistochemistry FGF-2 staining (red) in the kidney of a child with HIV-associated neuropathy (HIVAN) complicated by thrombotic microangiopathy (*n*=3 samples with HIVAN). Scale bars: 1.5 mm (C); 40 μm (D-F).

To determine the systemic clearance and ability of the kidneys to recruit circulating FGF-2, we injected [^125^I]-FGF-2 intravenously into WT and HIV-T_26_ mice with renal disease. As expected, most FGF-2 was rapidly cleared from the circulation within 6-12 min (Fig. 1A). However, more [^125^I]-FGF-2 was accumulated in the kidneys of HIV-Tg_26_ mice with renal disease, compared to WT mice (Fig. 1B). These changes were facilitated by the presence of a high number of FGF-2-binding sites in the kidneys of HIV-Tg_26_ with renal disease (Fig. 1C). In agreement with previous studies (Ray et al., 1994), FGF-2 was detected predominately in the peritubular interstitium of HIV-Tg_26_ mice with renal disease (Fig. 1D,E), where the FGF-2-binding sites were more significantly increased (Fig. 1C). In addition, as reported before (Ray et al., 2006), FGF-2 was detected in a similar location in children with HIV-CKDs (Fig. 1F).

### Circulating FGF-2 induces glomerular injury and albuminuria in WT and HIV-Tg_26_ mice

**Fig. 2.**
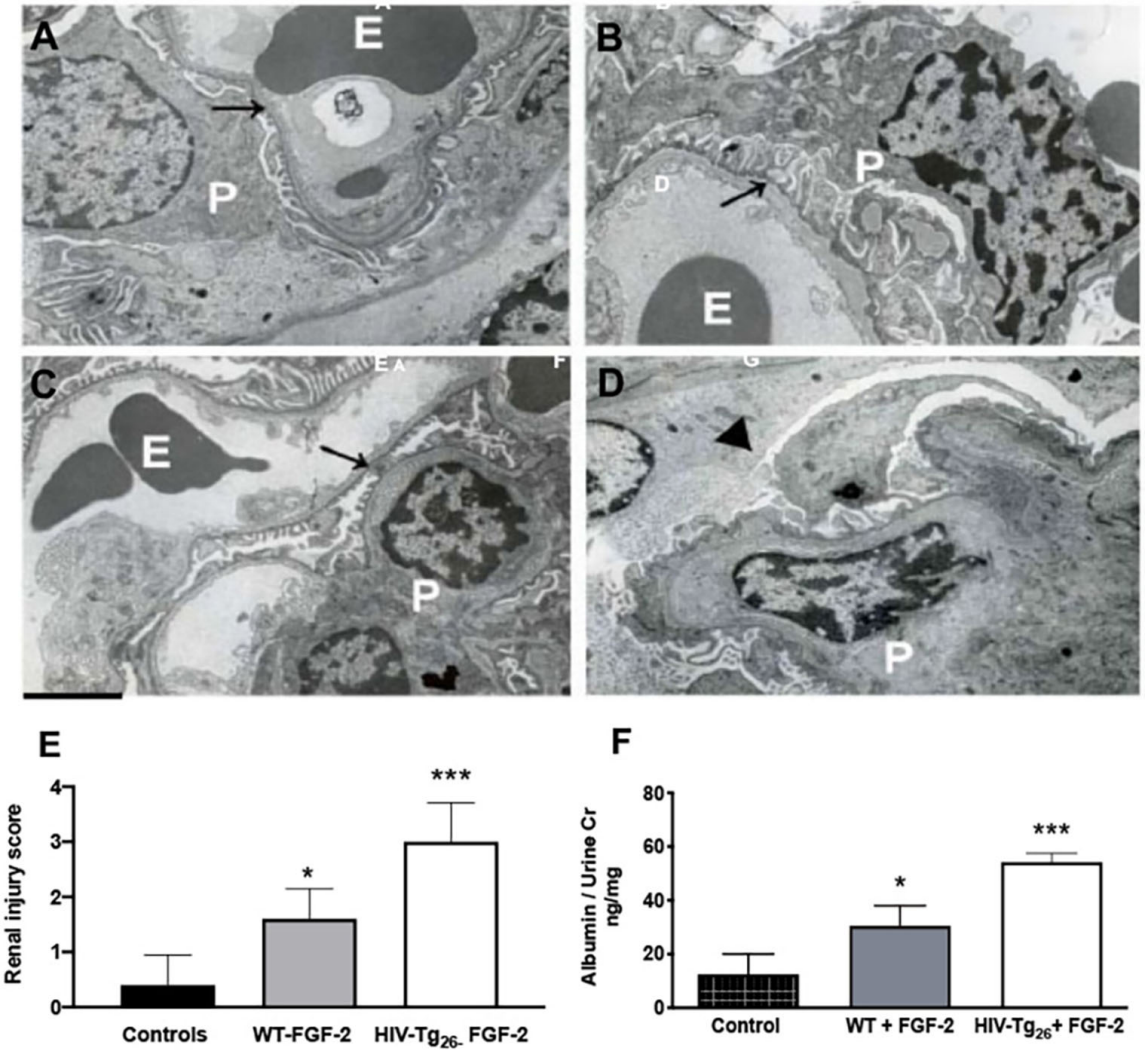
**Circulating FGF-2 induced glomerular ultrastructural lesions in WT and HIV-Tg_26_ mice.** A-D show representative electron microscopy pictures of renal glomeruli of WT and HIV-Tg_26_ mice injected daily intraperitoneally with either PBS control or recombinant human (rh) FGF-2 (10 μg/mouse/day×7 days). (A) Representative picture of normal glomerular endothelial cells and podocytes (arrow) from a WT mouse injected with PBS. E, erythrocytes; P, podocytes. (B) Representative picture of enlarged podocytes (arrow) and foot process effacement in an HIV-Tg_26_ mouse with HIVAN. (C) Representative picture of endothelial swelling (arrow) and mild podocyte foot process effacement from a WT mouse injected with rh-FGF-2. (D) Representative picture of a collapsed glomerular capillary (arrowhead), swollen endothelial cells and foot process effacement in an HIV-Tg_26_ mouse injected with rh-FGF-2. Scale bar: 2 μm (*n*=5 mice per group). (E) Renal injury scores generated as described in the Materials and Methods. WT mice injected with PBS served as controls. (F) Albuminuria in WT and HIV-Tg_26_ mice injected with rh-FGF-2. WT mice injected with PBS served as controls. The bars reflect the median and 95% confidence interval (CI) of five mice per group. ***P<0.001 and *P<0.05, compared to controls; one-way ANOVA (*n*=5 mice per group).

To determine whether FGF-2 recruited in the kidney was capable of inducing glomerular ultrastructural injury, we injected rh-FGF-2 intraperitoneally (i.p.) into 4-week-old WT and HIV-Tg_26_ male mice without pre-existing proteinuria (10 µg/mouse/day×7 days). By electron microscopy (EM), we found that FGF-2 induced endothelial swelling and mild fusion of the podocyte foot processes in WT mice (Fig. 2A,C). In HIV-Tg_26_ mice, FGF-2 induced more significant glomerular injury, mimicking the lesions seen in older HIV-Tg_26_ mice with HIVAN (Fig. 2B) and precipitating the collapse of glomerular capillaries (Fig. 2D). These glomerular ultrastructural changes were associated with dilated tubular structures, tubular casts, more severe renal injury scores (Fig. 2E) and albuminuria (Fig. 2F).

### Developing an FGF-2-inducible mouse model of childhood HIVAN

**Fig. 3.**
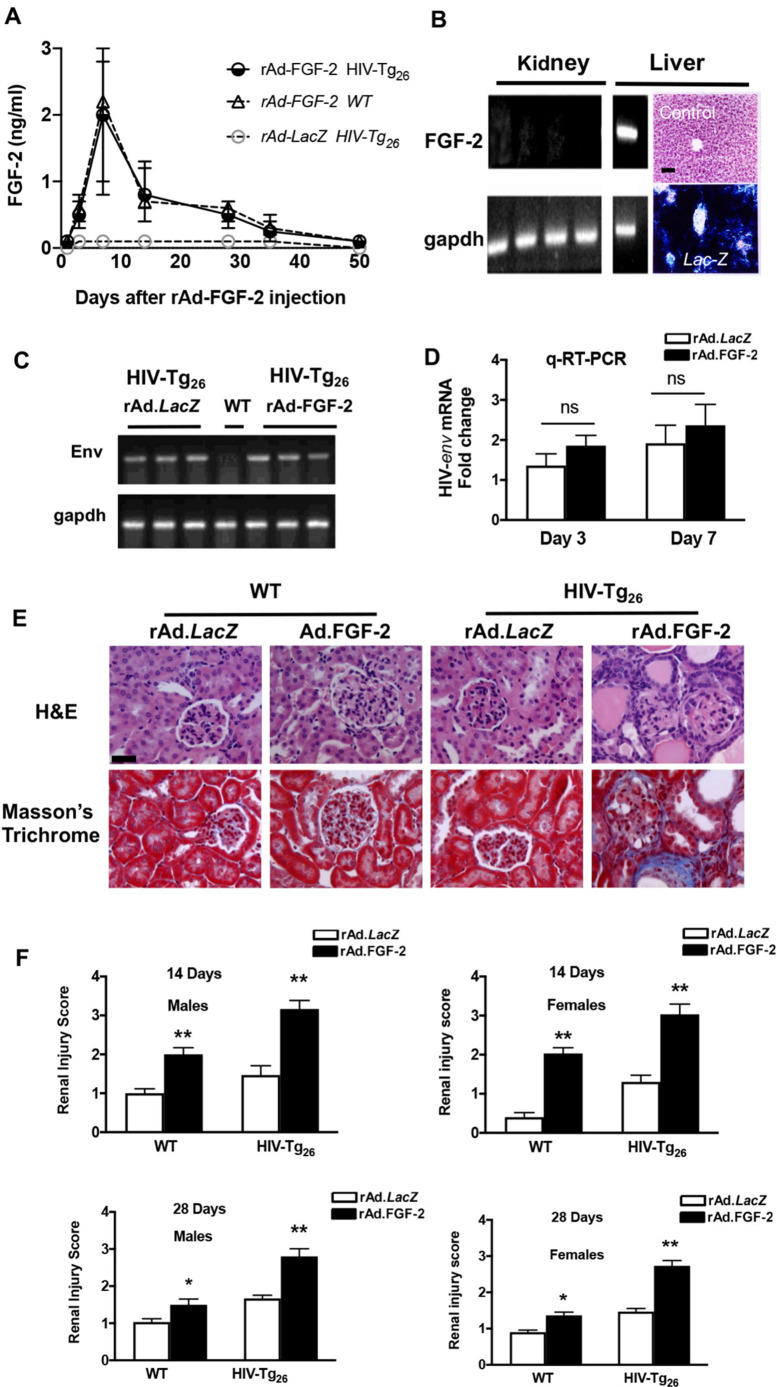
**An FGF-2 inducible mouse model of childhood HIVAN.** (A) Young WT and HIV-Tg_26_ male mice (3-week-old) were infected with recombinant adenoviral (rAd) vectors carrying a secreted form of human FGF-2 or rAd-*LacZ* control vectors, as described in the Materials and Methods (*n*=4 mice per group). Median and interquartile range FGF-2 values are shown for the corresponding days after the adenoviral injection. (B) Expression of human *FGF2* and mouse *Gapdh* mRNA by RT-PCR in the liver and kidneys of mice injected with rAd-FGF-2 vectors and harvested 7 days later. Human *FGF2* mRNA transcripts were detected only in the liver (*n*=4 mice per group). Mice injected with rAd-*LacZ* vectors revealed LacZ staining in the liver. In contrast, mice not injected with these vectors (control) showed no LacZ staining. Scale bar: 50 µm. (C) Expression of HIV envelope (*env*) and mouse *Gapdh* mRNA by RT-PCR in the kidney of HIV-Tg_26_ mice harvested 7 days after infection with either rAd-*LacZ* (controls) or rAd-FGF-2 vectors as described in the Materials and Methods. RNA extracted from a WT was used as a negative control. (D) Real-time quantitative RT-PCR analysis of HIV-*env* was performed in kidney RNA extracted from WT and HIV-Tg_26_ mice 3 and 7 days after the rAd-FGF-2 infections. Data are mean±s.e.m. (*n*=3 mice per group). Statistical significance was determined using a Mann–Whitney unpaired *t-*test. ns, non-significant (*P*>0.05). (E) Representative renal sections collected 14 days after the corresponding adenoviral injections from WT and HIV-Tg_26_ mice (*n*=5 mice per group). Scale bar: 20 µm. (F) The graphs represent the median and 95% CI renal injury scores derived from male and female WT and HIV-Tg_26_ mice 14 and 28 days after the corresponding adenoviral injections. Renal injury scores were generated as described in detail in the Materials and Methods. Statistical significance was determined using a Mann–Whitney unpaired *t-*test. *P<0.05 and **P<0.01, compared to the respective *LacZ* groups (*n*=4-6 mice per group).

To develop a new model of childhood HIVAN, we first assessed the natural history of renal disease in 95 young heterozygous HIV-Tg_26_ FBV/N mice from our inbred mouse colony during their first 2 months of life. Approximately 10% of these mice developed early proteinuria by 30 days of life and showed heavy proteinuria by the second month of life. Therefore, we screened all mice for the presence of proteinuria, and selected only 3- to 4-week-old HIV-Tg_26_ mice without abnormal proteinuria for all other studies. Because FGF-2 injected intravenously or i.p. is cleared from the circulation within minutes, we used recombinant adenoviral (rAd) vectors carrying a secreted form of human FGF-2 to sustain high plasma FGF-2 levels for several days (Fig. 3A). This approach mimicked the situation seen in children with a high viral load, who frequently maintain high plasma FGF-2 levels for months or years (Ray et al., 1999). As expected, based on our previous studies (Ye et al., 2000), the systemic injection of adenoviral vectors via the retro-orbital venous plexus in 3- to 4-week-old mice resulted in the transduction of hepatocytes. Human *FGF2* mRNA transcripts were detected in the liver, but not in the kidney (Fig. 3B). In a similar manner, LacZ staining was detected in the liver of mice injected with rAd-*LacZ* vectors (Fig. 3B). Therefore, in this mouse model, hepatocytes transduced with the rAd-FGF-2 vectors released FGF-2 into the circulation. In both WT and HIV-Tg_26_ mice, the peak plasma FGF-2 levels were reached ∼7 days after the rAd-FGF-2 injection, and decreased slowly until the FGF-2 plasma levels became undetectable by ∼35 days (Fig. 3A) due to the clearance of the rAd vectors by the immune system. As expected, HIV-Tg_26_ mice injected with rAd-*LacZ* vectors showed no detectable plasma levels of human FGF-2 (Fig. 3A). We found no significant differences in the expression levels of HIV-*env* mRNA in the kidney of HIV-Tg_26_ mice injected with the corresponding FGF-2 or *LacZ* adenoviral vectors (Fig. 3C,D). In total, we injected 53 male and 42 female mice with the control and FGF-2 viral vectors, including WT and HIV-Tg_26_ mice, and found that FGF-2 precipitated HIVAN within 2-3 weeks in HIV-Tg_26_ mice (Fig. 3E,F). WT mice injected with rAd-FGF-2 developed less-severe and transient HIVAN-like lesions (Fig. 3E,F), despite having similar levels of circulating FGF-2 (Fig. 3A). Finally, we found no significant differences between the renal injury scores of male and female mice injected with rAd-FGF-2 (Fig. 3F).

#### Glomerular proliferative changes in WT and HIV-Tg_26_ mice

**Fig. 4.**
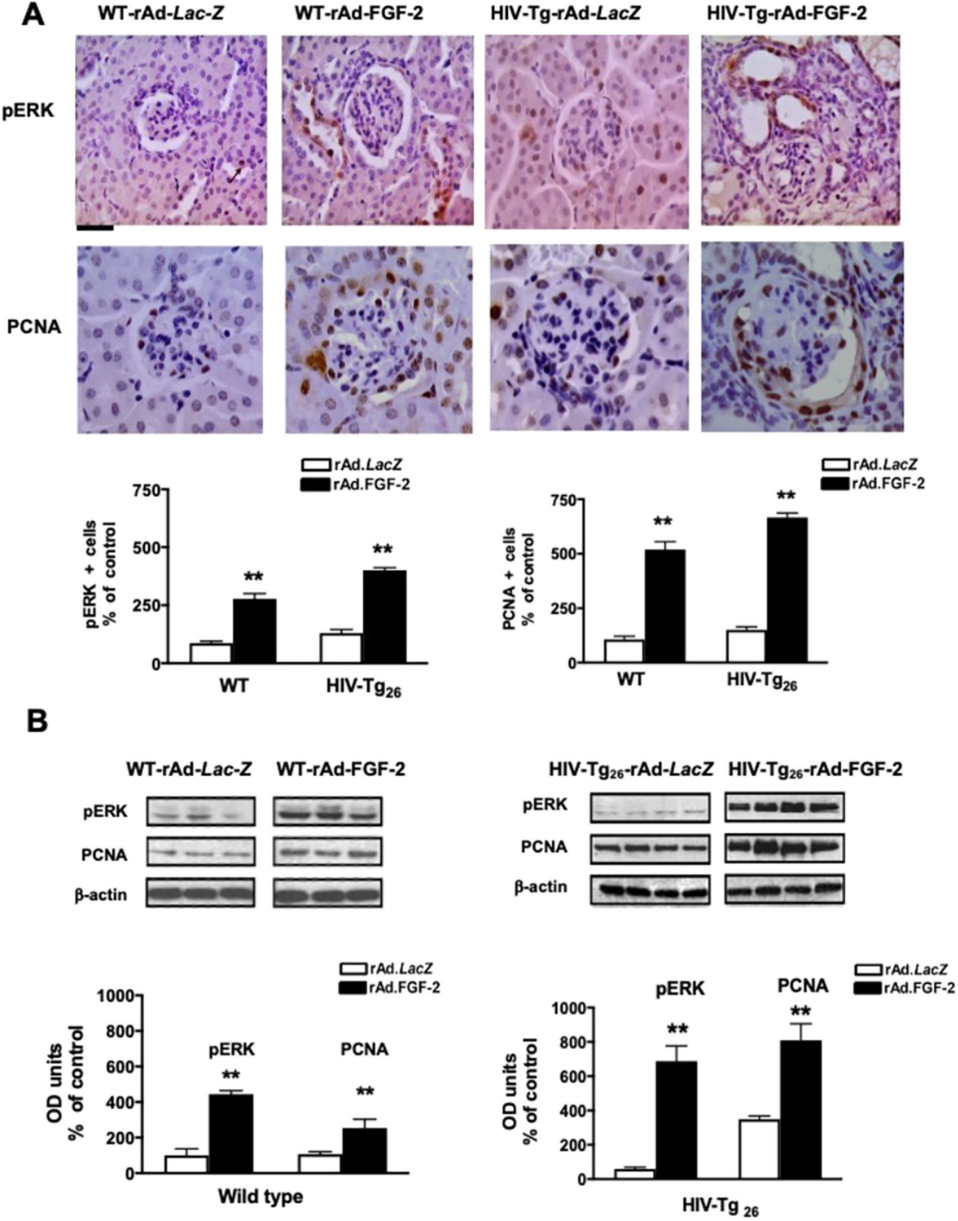
**rAd-FGF-2 induced the expression of pERK and PCNA in podocytes and tubular epithelial cells.** (A) Representative immunohistochemistry staining for pERK and PCNA (both brown) in renal sections harvested from WT and HIV-Tg_26_ mice 14 days after the corresponding adenoviral injections. The graphs represent percentage changes in the number of positive cells (mean±s.e.m.) relative to the corresponding control groups. **P<0.01, Mann–Whitney unpaired *t-*test (*n*=3-4 mice per group). Scale bar: 20 µm. (B) Representative results of the western blot analysis for pERK and PCNA performed in kidney homogenates derived from WT and HIV-Tg_26_ mice harvested 14 days after infection with the corresponding adenoviral vectors. The expression of pERK and PCNA was quantitated as a ratio of β-actin. The graphs show the results of the densitometry analysis and quantification of the results in optical density (OD) units (mean±s.e.m.), as described in the Materials and Methods. Statistical significance was determined using a Mann–Whitney unpaired *t-*test. **P<0.01, compared to WT or HIV-Tg_26_ mice infected with rAd-*LacZ* vectors (*n*=3-4 mice per group).

**Fig. 5.**
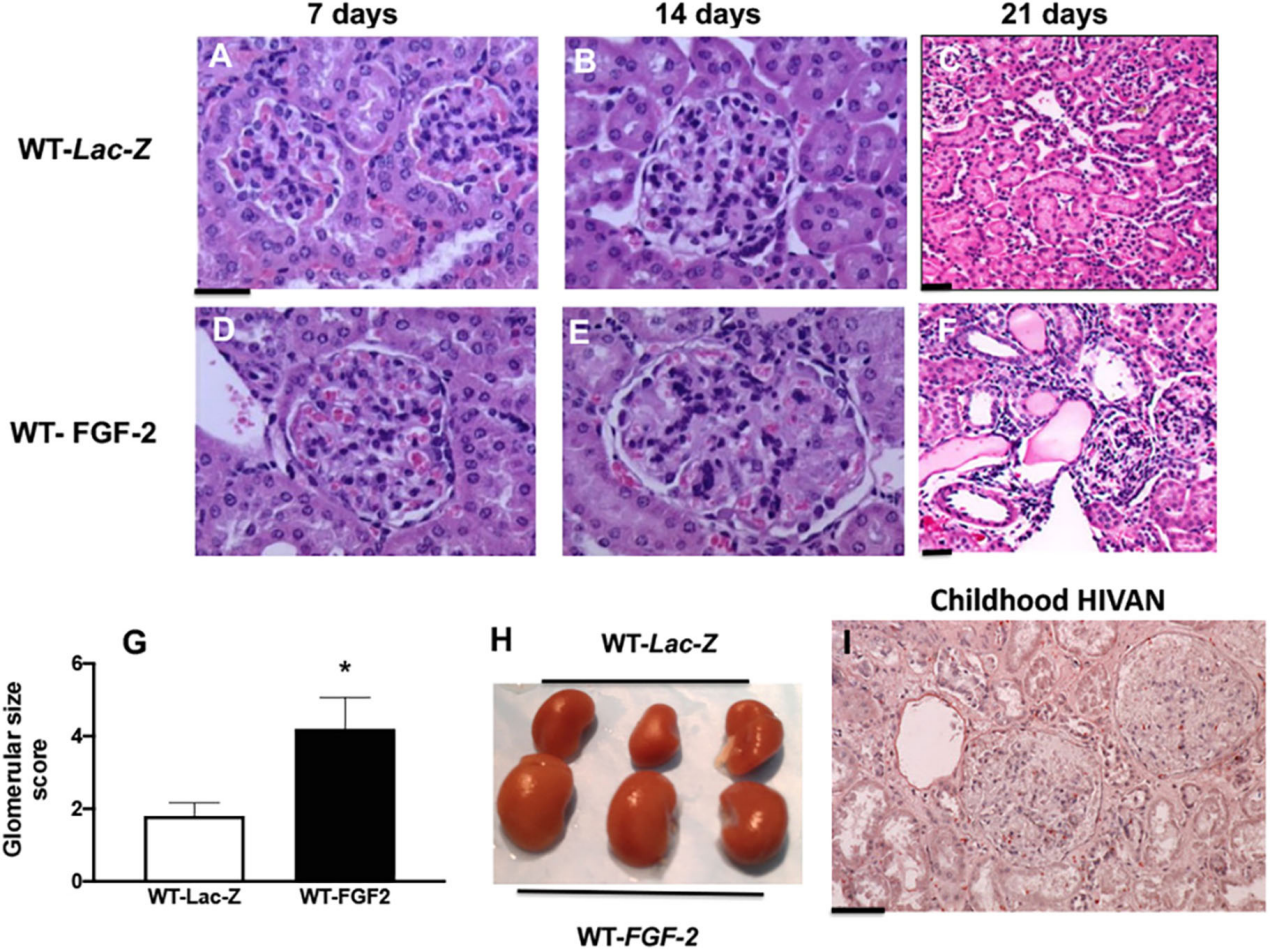
**Sustained high plasma levels of FGF-2 increase the glomerular size of WT mice infected with rAd-FGF-2 vectors.** (A-F) Representative renal sections harvested from WT mice infected with rAd-*LacZ* or rAd-FGF-2 vectors and stained with Hematoxylin and Eosin (*n*=5 mice per group). Scale bars: 20 µm. (G) The graph shows the mean±s.d. values corresponding to the glomerular size scores assessed as described in the Materials and Methods. *P<0.05, Mann–Whitney unpaired *t-*test (*n*=5 mice per group). (H) Kidneys taken from three different WT mice injected with rAd-FGF-2 and selected based on their high plasma FGF-2 levels 21 days after the injection were enlarged, compared to those from WT mice injected with rAd-*LacZ*. (I) A representative renal section from a child with HIVAN stained with Hematoxylin shows two enlarged glomeruli (*n*=3 samples with HIVAN). Scale bar: 20 µm.

Three-week-old WT and HIV-Tg_26_ mice injected with rAd-FGF-2 vectors developed proliferative changes in glomerular and tubular epithelial cells. Briefly, immunohistochemistry and western blot studies revealed that the expression levels of phosphorylated extracellular signal-regulated kinase (pERK) and the proliferating cell nuclear antigen (PCNA) were elevated in all mice injected with rAd-FGF-2 vectors, compared to those injected with the control *LacZ* vectors (Fig. 4). Because children with HIVAN usually showed mesangial hyperplasia and enlarged glomeruli, we explored whether FGF-2 was capable of inducing these changes in WT mice and found that FGF-2 increased the size of renal glomeruli in these mice (Fig. 5A-H). Furthermore, kidneys harvested from a selected group of WT mice showing high FGF-2 plasma levels after 21 days were significantly enlarged, compared to those taken from WT mice injected with rAd-*LacZ* vectors (Fig. 5H). Taken together, these findings suggest that FGF-2 can induce many histological features characteristic of childhood HIVAN (Fig. 5I), including increased glomerular size (Fig. 5D-F), tubular dilatation with casts (Fig. 5F) and even overall renal enlargement in a few selected cases (Fig. 5H). Nonetheless, as discussed above, these changes improved spontaneously when the plasma levels of human FGF-2 became undetectable after 28 days. Taken together, these findings suggest that circulating FGF-2 needs to act in synergy with HIV transcripts expressed in kidney cells in order to precipitate the full HIVAN phenotype and HIV-CKD in a short period of time.

#### De-differentiation of podocytes in HIV-Tg_26_ mice infected with rAd-FGF-2 vectors

**Fig. 6.**
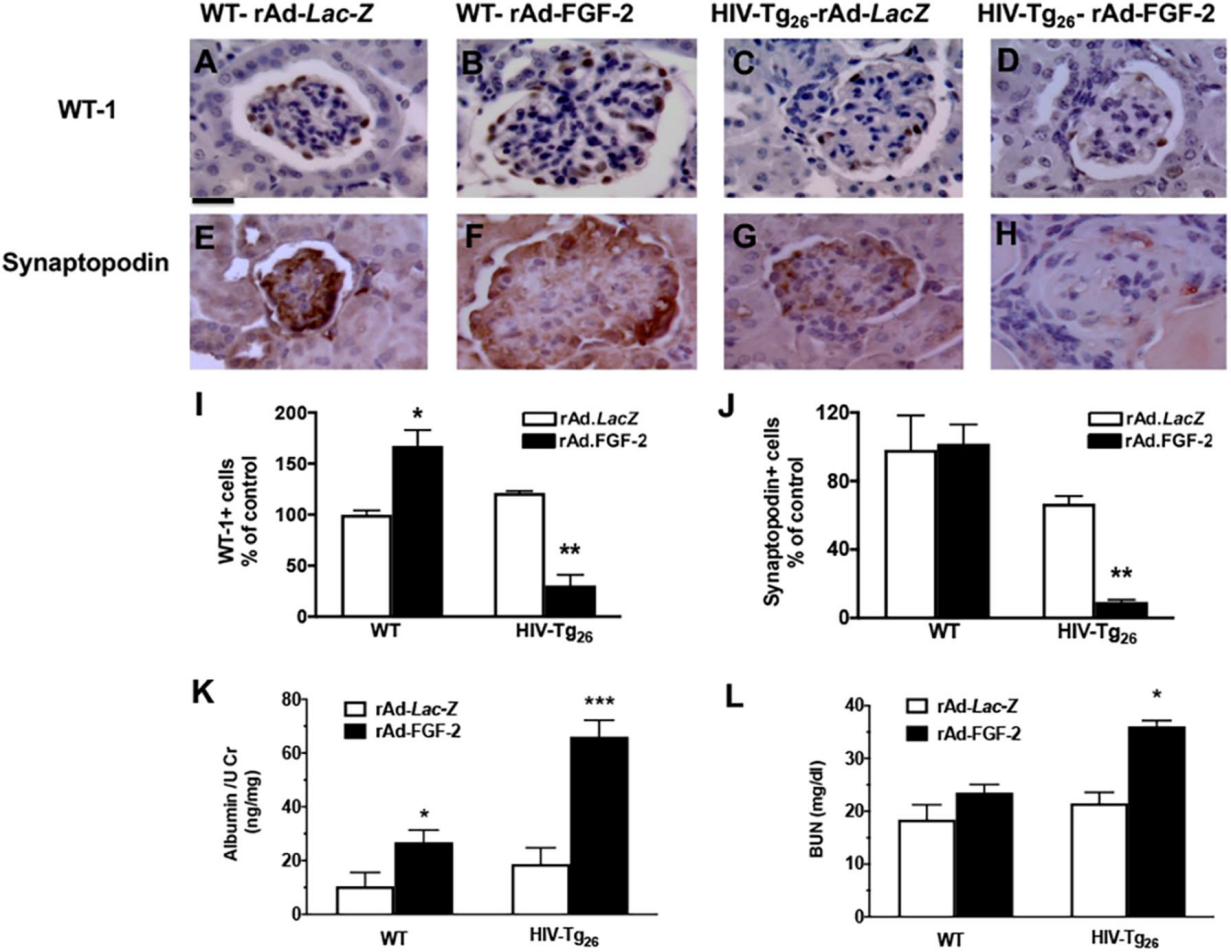
**rAd-FGF-2 induced de-differentiation changes in podocytes and albuminuria and increased the blood urea nitrogen (BUN) levels of HIV-Tg_26_ mice.** (A-H) Representative immunohistochemistry staining for WT1 and synaptopodin (both brown in renal sections harvested 14 days after the adenoviral injections) (*n*=4-5 mice per group). Scale bar: 20 µm. (I,J) Percentage changes in WT1^+^ (I) and synaptopodin^+^ (J) cells relative to the corresponding control groups (mean±s.e.m.; *n*=4-5 mice per group). (K) WT and HIV-Tg_26_ mice injected with rAd-FGF-2 vectors developed significant albuminuria compared to the corresponding control groups (*n*=4-5 mice per group). (L) HIV-Tg_26_ mice showed elevated BUN levels 28 days after the injection of rAd-FGF-2 vector (*n*=4-5 mice per group). Statistical significance was determined using a Mann–Whitney unpaired *t-*test. *P<0.05, **P<0.01 and ***P<0.001, compared to the corresponding *LacZ* groups.

Because the pathogenesis of HIVAN is characterized by the loss of podocyte differentiation markers such as WT1 and synaptopodin, we carried out immunohistochemistry studies in kidney sections derived from WT and HIV-Tg_26_ mice to assess these changes. We found an increased number of WT1^+^ glomerular cells in WT mice injected with rAd-FGF-2 vectors, compared to those injected with rAd-*LacZ* vectors (Fig. 6A,B,I). However, the number of synaptopodin^+^ cells did not change significantly between these groups (Fig. 6E,F,J). In contrast, the number of WT1^+^ and synaptopodin^+^ cells decreased in HIV-Tg_26_ mice injected with rAd-FGF-2 vectors (Fig. 6C,D,G-J). Although both WT and HIV-Tg_26_ mice infected with rAd-FGF-2 vectors developed significant albuminuria (Fig. 6K), only HIV-Tg_26_ mice injected with rAd-FGF-2 vectors developed glomerular sclerotic lesions (Fig. 6D,H) and showed high blood urea nitrogen (BUN) levels after 28 days (Fig. 6L).

#### FGF-2 induces tubulo-interstitial lesions in HIV-Tg_26_ mice that mimic those seen in childhood HIVAN

**Fig. 7.**
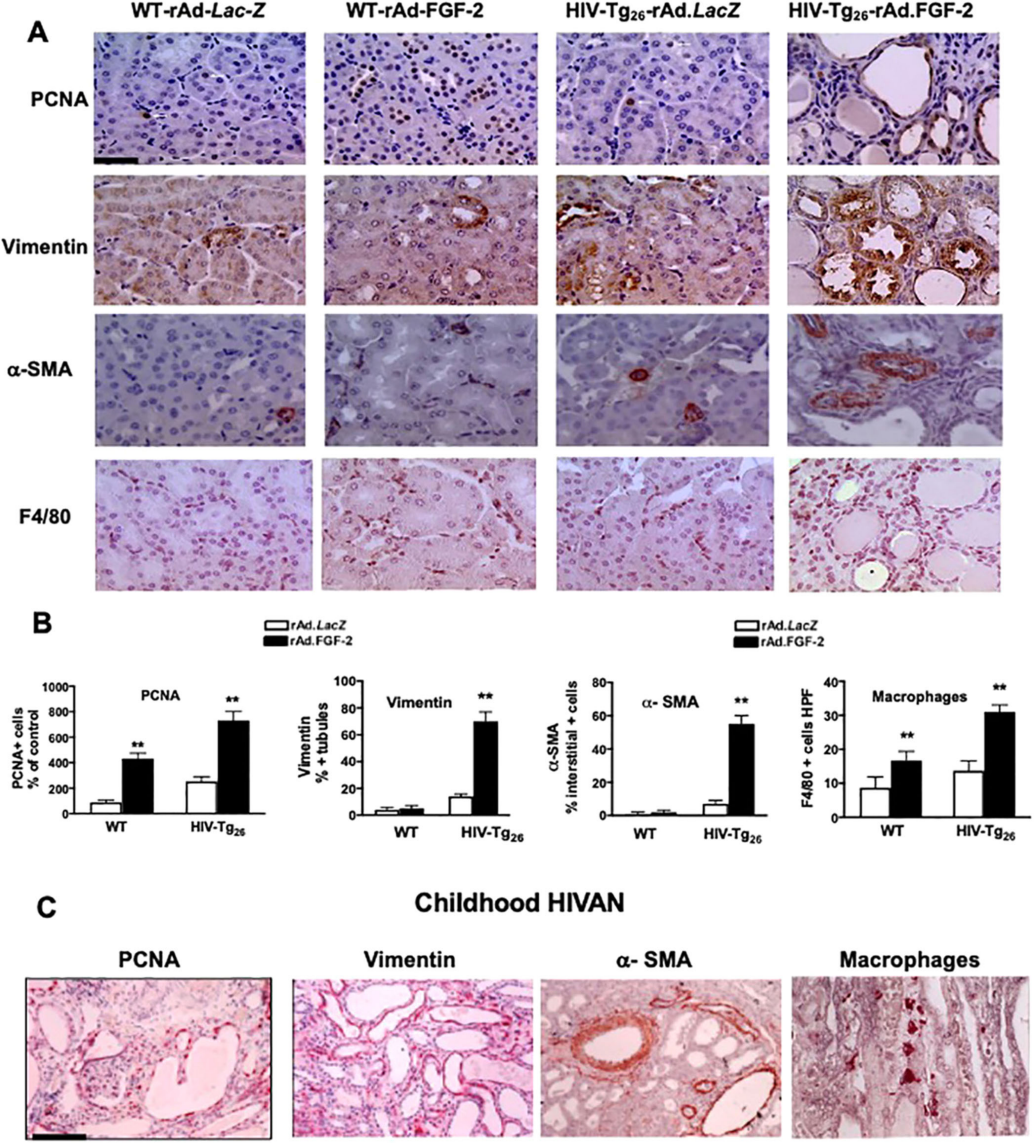
**Circulating FGF-2-induced tubulo-interstitial changes in HIV-Tg_26_ mice and mimic those seen in childhood HIVAN.** (A) Representative immunohistochemistry staining for PCNA^+^, vimentin^+^ (both brown), α-smooth muscle actin^+^ (α-SMA^+^) and F4/80^+^ macrophages (both red) in renal sections harvested from WT and HIV-Tg_26_ mice 14 days after the injections of rAd-*LacZ* (control) or rAd-FGF-2 vectors (*n*=3-4 mice per group). Scale bar: 20 µm. (B) Quantification scores (mean±s.e.m.) relative to the respective rAd-*LacZ* control groups. Statistical significance was determined using the Mann–Whitney unpaired *t-*test. **P<0.01, compared to the corresponding rAd-*LacZ* group (*n*=3-4 mice per group). (C) Representative immunohistochemistry staining for PCNA^+^, vimentin^+^, α-SMA^+^ and CD68^+^ macrophages in renal sections taken from children with HIVAN (*n*=3 samples with HIVAN). Scale bar: 20 µm.

As shown by immunohistochemistry, HIV-Tg_26_ mice injected with rAd-FGF-2 vectors developed significant tubulo-interstitial proliferative lesions and pro-fibrotic inflammatory changes (Fig. 7A,B). More specifically, we explored the changes in markers of cell proliferation (PCNA), de-differentiation (vimentin), inflammation (F4/80) and fibrosis [α-smooth muscle actin (α-SMA)] that are affected in childhood HIVAN. The expression levels of PCNA and vimentin in renal tubular epithelial cells (Fig. 7A), as well as the number of α-SMA- and F4/80-expressing cells, were all increased in the renal interstitium of HIV-Tg_26_ mice injected with rAd-FGF-2 vectors (Fig. 7A; Fig. S1). These changes mimic those seen in children with HIVAN in a remarkable manner (Fig. 7C). Furthermore, WT mice injected with rAd-FGF-2 vectors showed a higher number of renal tubular epithelial cells expressing PCNA^+^ and interstitial F4/80^+^ macrophages, compared to control mice injected with rAd-*LacZ* (Fig. 7A,B). The latter findings suggest that FGF-2 can promote the renal recruitment of HIV^+^ inflammatory cells, which in turn facilitates the infection of kidney epithelial cells in children living with HIV. In summary, the glomerular and tubulo-interstitial changes seen in HIV-Tg_26_ mice injected with rAd-FGF-2 vectors were similar to those seen in HIV-Tg_26_ mice that developed HIVAN spontaneously (Fig. S1) and in children with HIVAN (Fig. 7C).

#### The FGF/VEGF receptor tyrosine kinase inhibitor PD173074 improves the outcome of HIV-Tg_26_ mice infected with rAd-FGF-2 vectors

**Fig. 8.**
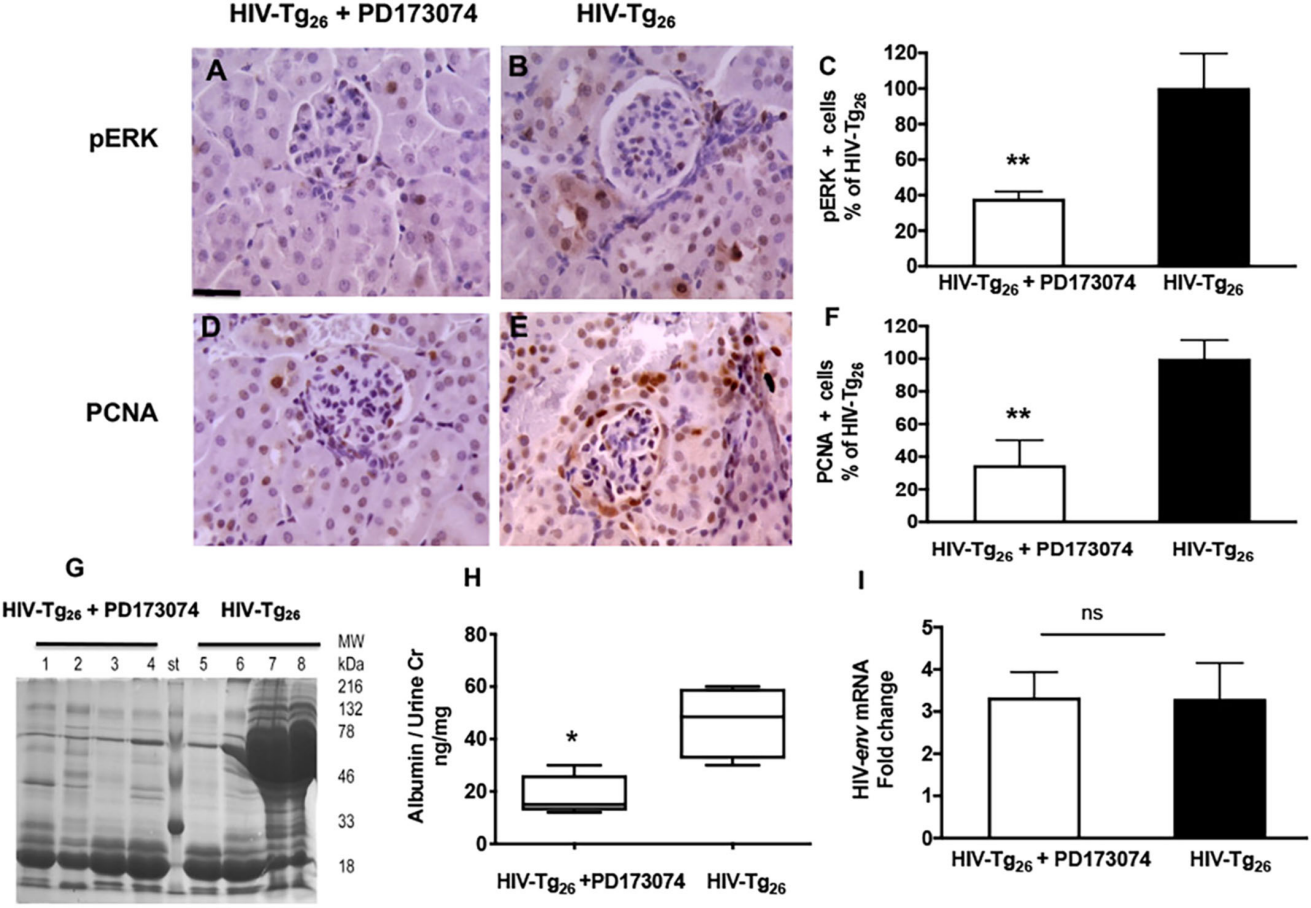
**The FGF/VEGF receptor tyrosine kinase inhibitor PD173074 improves the outcome of the circulating FGF-2-induced HIVAN in HIV-Tg_26_ mice.** (A,B,D,E) Representative immunohistochemistry staining for pERK and PCNA (both brown) in renal sections harvested from HIV-Tg_26_ mice infected with rAd-FGF-2 vectors and treated with the FGF/VEGF tyrosine kinase inhibitor PD173074 or control vehicle (PBS-10% DMSO) as described in the Materials and Methods (*n*=4-5 mice per group). Scale bar: 20 µm. (C,F) Percentage changes in pERK^+^ and PCNA^+^ cells (mean±s.e.m.) between the two groups. **P<0.01, Mann–Whitney *t-*test (*n*=4-5 mice per group). (G) Coomassie Blue-stained SDS-PAGE gel loaded with urine samples (5 µl) collected at the end of the experiment (*n*=4 mice per group). (H) Albuminuria was quantified as described in the Materials and Methods, and expressed as a ratio of the urinary creatinine. The box-and-whisker plots show the minimum and maximum values. *P<0.05, Mann–Whitney unpaired *t-*test (*n*=4-5 mice per group). (I) Real-time RT-PCR analysis of HIV-*env* was performed in kidney RNA extracted from both groups of mice at the end of the treatment. Data are mean±s.e.m. (*n*=4 mice per group). Statistical significance was determined using a Mann–Whitney unpaired *t-*test. ns, non-significant (*P*>0.05).

As described above, the most consistent pathological findings seen in HIV-Tg_26_ mice infected with rAd-FGF-2 were the glomerular and tubular proliferative lesions associated with heavy proteinuria. Thus, to determine whether these changes could be reversed by blocking the pERK pathway, HIV-Tg_26_ mice infected with rAd-FGF-2 vectors were treated with the FGF/VEGF tyrosine kinase inhibitor PD17307, which blocks the pERK pathway. Briefly, we found that PD179374 reduced the expression of pERK and PCNA in renal glomerular and tubular epithelial cells (Fig. 8A-F) and ameliorated the proteinuria (Fig. 8G,H), without affecting the kidney expression of HIV genes (HIV-*env*) (Fig. 8I). In summary, these findings support the notion that circulating FGF-2 can precipitate HIVAN in HIV-Tg_26_ mice, at least partially, by inducing the pERK pathway in kidney cells.

## DISCUSSION

In this study, we present new evidence to demonstrate that circulating FGF-2 precipitates HIVAN in young HIV-Tg_26_ mice by activating the pERK pathway in glomerular and tubular epithelial cells without previously inducing the expression of HIV-1 genes. In addition, we developed a new FGF-2-inducible mouse model system of childhood HIVAN that reproduces the full HIVAN phenotype, and could be used to test new therapies to prevent the progression of this disease in children.

To define the role of circulating FGF-2 in childhood HIVAN, we utilized HIV-Tg_26_ mice and their corresponding WT littermates. These mice have been described in detail in previous studies (Kopp et al., 1992; Dickie et al., 1991). Briefly, they carry a 7.4 kb HIV-1 construct lacking a 3 kb sequence overlapping the *gag*/*pol* region of the HIV provirus pNL4-3 (Felser et al., 1989), and express HIV genes in the kidney and other tissues (Kopp et al., 1992). Most heterozygous HIV-Tg*_2_*_6_ mice develop HIVAN spontaneously at different time points, approximately by 45-90 days of life (Kopp et al., 1992). Therefore, in order to develop a reliable and cost-effective mouse model of childhood HIVAN, it is necessary to induce HIVAN in a synchronized fashion during the first month of life. Considering that high plasma and urine levels of FGF-2 have been detected in most children with HIVAN (Ray et al., 1999; Soler-Garcia et al., 2009), we hypothesized that circulating FGF-2 plays a relevant role precipitating childhood HIVAN. To test this hypothesis, we injected rh-FGF-2 daily into young WT and HIV-Tg_26_ mice for 7 days, and also used adenoviral vectors carrying a secreted form of rh-FGF-2 (Mattison et al., 2012) to maintain the high plasma FGF-2 levels for weeks. Overall, we found that circulating FGF-2 induced many of the renal histological features typical of childhood HIVAN in young WT mice and precipitated HIVAN within ∼14 days in HIV-Tg_26_ mice. More specifically, FGF-2 increased the size of renal glomeruli and induced the proliferation of renal epithelial cells, decreased the expression of the podocyte differentiation markers WT1 and synaptopodin in HIV-Tg_26_ mice, and precipitated the development of proteinuria, tubular dilatation and renal inflammatory changes in all mice. In particular, the expression levels of vimentin^+^, α-SMA^+^ and F4/80^+^ macrophages were all increased in the tubulo-interstitium of HIV-Tg_26_ mice injected with rAd-FGF-2 vectors. These findings suggest that circulating FGF-2 can precipitate all the pathological features characteristic of childhood HIVAN in young HIV-Tg_26_ mice.

FGF-2 is a heparin-binding growth factor that lacks a conventional signal sequence for secretion and is stored as an ‘inactive pool’ bound to heparan sulfate proteoglycans in the vessel walls, basement membranes and extracellular matrix, where it remains protected from proteolytic degradation (Bashkin et al., 1989). Therefore, very-low plasma levels of FGF-2 are detected in the circulation of healthy children (Ray et al., 2002). In the young human kidney, FGF-2 is detected in the Bowman's capsule, renal vessels, basement membranes and tubules (Gonzalez et al., 1996), and plays an important role during renal development (Bates, 2011) and regeneration after injury (Gupta et al., 2000; Wai et al., 2013; Ray et al., 2002; Xu et al., 2020). However, FGF-2 can also be released through non-conventional pathways by injured endothelial cells, cytokines and proteases (D'Amore, 1990) that are produced by HIV-infected cells (Samaniego et al., 1995; Ascherl et al., 2001). These findings explain why both children and adults (Ascherl et al., 2001) living with a high viral load have high plasma FGF-2 levels. In addition, we showed that the kidney of HIV-Tg_26_ mice with renal disease can act as a ‘sink’, trapping circulating FGF-2. In this manner, FGF-2 accumulated in the kidney can stimulate the proliferation of renal epithelial cells. Because podocytes are terminally differentiated cells that are unable to undergo cell division, it is tempting to speculate, as suggested before (Sasaki et al., 1999, 1997), that forcing the proliferation of podocytes may precipitate their detachment and/or death. In agreement with this notion, daily injections of FGF-2 to normal rats or monkeys for up to 60-120 days caused podocyte injury and glomerulosclerosis (Kriz et al., 1995; Mazue et al., 1993).

Our findings provide an alternative explanation to understand why it is difficult to prevent the long-term progression of well-established childhood HIVAN despite modern antiretroviral treatments (Beng et al., 2020). Briefly, one current pathological paradigm for HIVAN is that HIV-1 induces a productive infection of podocytes (Marras et al., 2002), generating HIV transcripts (e.g. *nef*) that induce the de-differentiation and proliferation of these cells directly (He et al., 2004). However, given the limited number of renal epithelial cells that appear to be productively infected in human renal biopsies, as well as their focal pattern of distribution (Marras et al., 2002; Bruggeman et al., 2000), it is unlikely that the widespread epithelial proliferative changes characteristic of childhood HIVAN are caused directly by HIV-1 genes. Our model supports the notion that circulating FGF-2 can induce the proliferation of renal epithelial cells and precipitate HIVAN in HIV-Tg_26_ without upregulating the expression of HIV genes. Furthermore, expression of the APOL1 risk variants in cultured human podocytes (Chun et al., 2019), or *APOL1* transgenic mice (Beckerman et al., 2017; Aghajan et al., 2019), can also precipitate their injury or death. In addition, HIV-infected cells release interferon-γ and TNF-α, and both cytokines are capable of inducing the release of FGF-2 (Samaniego et al., 1995; Ascherl et al., 2001) and increasing the expression levels of APOL1 in podocytes (Nichols et al., 2015; Aghajan et al., 2019). Taken together, these data suggest that HIV-1, alone or in combination with the APOL1 risk variants, induces podocyte injury, rather than direct proliferative changes, in human podocytes. In support of this notion, we have shown that HIV-1 can infect and injure podocytes cultured from children with HIVAN via a transmembrane-TNF-α-dependent mechanism that involves clathrin-mediated endocytosis and NF-κB activation (Li et al., 2017). Thus, it is possible that podocytes expressing HIV transcripts may be more sensitive to the cytotoxic effects of the *APOL1* risk alleles and the mitogenic effects of FGF-2. This alternative pathologic paradigm can explain why almost all primary renal epithelial cells cultured from the urine of children with HIVAN do not express HIV transcripts and why HIV^+^ renal epithelial cells cannot be expanded in tissue culture (Ray et al., 1998a).

FGF-2 binds to different FGF receptors and initiates signaling through mitogen-activated protein kinases (MAPKs) (Tian et al., 2000; Cobb et al., 1991). pERK is a major downstream mediator of FGF-2/FGFR2 signaling, and is essential to mediate FGF-2-induced proliferation, migration and differentiation. In this manner, pERK plays important roles during renal development (Bates, 2011) and in several pediatric kidney diseases, including acute kidney injury (Xu et al., 2020) and polycystic kidney disease (Liang et al., 2008). In previous studies, we reported that FGF-2 activated the pERK pathway and induced cysts in the kidneys of newborn mice (Li et al., 2006). We also found that FGF-2 induced the pERK pathway in primary renal epithelial cells cultured from the urine of children with HIVAN (Izevbigie et al., 2000). In the current study, we found that the FGF/VEGF receptor tyrosine kinase inhibitor PD173074 was capable of inhibiting the pERK pathway, and reducing the proteinuria and proliferative changes in HIV-Tg_26_ mice injected with rAd-FGF-2 vectors. These findings are in agreement with the notion that other viral proteins and heparin-binding growth factors that are involved in the pathogenesis of HIVAN (e.g. Nef, Tat, VEGF-A) (He et al., 2004; Das et al., 2016; Korgaonkar et al., 2008; Tang et al., 2020) also activate the pERK pathway and can act in synergy with FGF-2 (Seghezzi et al., 1998). Nonetheless, PD170374 did not completely normalize the proteinuria or completely reverse the renal histological lesions in HIV-Tg_26_ mice injected with rAd-FGF-2 vectors. Therefore, other pathways should be involved in this process, and further studies are needed to identify them.

Finally, we should acknowledge the limitations of the FGF-2-inducible mouse model of childhood HIVAN. The most relevant one is that the plasma levels of FGF-2 during the first 2 weeks after the adenoviral injections are ∼10-fold higher than those seen in children with HIVAN (Ray et al., 1999). However, all mice were followed for ∼28-35 days, until the plasma FGF-2 levels became undetectable. In contrast, children living with a high viral load usually maintain high plasma FGF-2 levels for several months or years (Ray et al., 1999). In addition, children with HIV-CKD showed upregulated expression of the renal FGF-2-binding sites (Ray et al., 2004), which facilitate the recruitment of circulating FGF-2. Thus, the plasma FGF-2 levels are unlikely to reflect the concentration of FGF-2 in the kidneys, in particular, considering that FGF-2 is cleared from the circulation within minutes (Whalen et al., 1989). Another limitation of this model is that mice do not express APOL1 risk variants. Nonetheless, this issue can be solved by generating dual HIV-Tg_26_ mice carrying *APOL1* transcripts, as was done by others (Bruggeman et al., 2019). In addition, many children develop HIVAN independently of the APOL1 risk variants (Purswani et al., 2016; Ekulu et al., 2019). Finally, young mice are more sensitive to the renal mitogenic effects of FGF-2 (Li et al., 2006), and children have higher plasma levels of FGF-2 compared to adults. Therefore, this model may be less relevant to explore the pathogenesis of HIVAN in adults.

In conclusion, we have developed a new mouse model of childhood HIVAN and showed that circulating FGF-2 precipitated this disease in HIV-Tg_26_ mice by inducing the pERK pathway without previously increasing the renal expression of HIV genes. When these findings are taken into consideration in the context of previous studies, they suggest that circulating FGF-2 may be an independent risk factor for precipitating HIVAN and other HIV-CKDs in children.

## MATERIALS AND METHODS

### HIV-Tg_26_ mice

This study was approved by the Children's Research Institute Animal Care and Use Committee. All mice used in this study had free access to water and standard food, and were treated in accordance with the National Institutes of Health guidelines for the care and use of research animals. We used heterozygous HIV-Tg_26_ FVB/N transgenic mice (Dickie et al., 1991) [carrying a 7.4 kb non-infectious clone of the pNL4-3 provirus lacking a 3 kb sequence overlapping the *gag* and *pol* genes, but including the 5′ and 3′ long-terminal repeats and the *env*, *tat*, *nef*, *rev*, *vif*, *vpr* and *vpu* HIV genes (Felser et al., 1989)] and their WT littermates. The natural history of HIV-Tg_26_ mice has been described in detail in previous studies (Dickie et al., 1991; Kopp et al., 1992).

### Injections of rh-FGF-2

Endotoxin free rh-FGF-2 was purchased from R&D Systems (AFL233)*.* Ten 3-week-old WT or HIV-Tg_26_ FVB/N male mice without pre-existing abnormal proteinuria were divided in two groups of five mice each, and injected i.p. with FGF-2 (10 μg daily for 7 consecutive days) or phosphate-buffered saline (PBS) vehicle. All mice were euthanized by cervical dislocation under halothane anesthesia 7 days after the first injection. Renal sections were harvested and processed for light microscopy and EM as described previously (Ray et al., 1994). Renal injury scores (1-4) were determined blindly by examining five randomly selected EM sections per mouse, and also by light microscopy, counting the percentage of glomerular and tubular structures exhibiting segmental or global sclerosis, tubular dilatation/casts, microcysts and inflammation as discussed in detail in the ‘Renal injury scores’ section below.

### Recruitment of circulating FGF-2 and FGF-2 binding studies

FVB/N WT or HIV-Tg_26_ male mice (*n*=3 per group) were anesthetized with ketamine and xylazine (70-7 mg/kg body weight) and injected intravenously with 0.1 ng [^125^I]-FGF-2 diluted in 100 μl normal saline (specific activity 1 μCi/ng). Subsequently, 20 μl blood was collected from the left eye by retro-orbital bleeding at different time points, and all mice were euthanized by cervical dislocation under anesthesia to remove the kidneys. The radioactivity of the blood and kidney samples was measured with a gamma counter as previously described. The [^125^I]-FGF-2 binding studies were done by autoradiography as described previously (Ray et al., 1994). Briefly, frozen renal sections (16 μm) were preincubated for 15 min in binding buffer [Dulbecco's modified Eagle medium (DMEM), 20 mM HEPES at pH 7.4, and 0.15% gelatin] and exposed to 0.25 nM [^125^I]-FGF-2 at 4°C for 2 h. Nonspecific binding was determined by displacing the binding of [^125^I]-FGF-2 with 300 μg/ml heparin as described previously (Ray et al., 1994). All slides were washed, dried and exposed to an autoradiography film inside a cassette under similar conditions at room temperature for up to 4 days. Differences in binding were determined by counting the optical density of the autoradiograms in specific kidney areas of 0.22 mm^2^ using a computerized program as described previously (Ray et al., 1994).

### Adenoviral vectors

The generation of the rAd-FGF-2 and rAd-*LacZ* vectors was described in detail in previous studies. Both FGF-2 and LacZ control adenoviruses were amplified, purified, desalted and titrated as described previously (Kozarsky et al., 1993; Jerebtsova et al., 2007). The rAd-FGF-2 vectors carry a 700 bp cDNA sequence encoding a secreted form of human FGF-2 (rAd-FGF-2) (Gupta et al., 2001), while the rAd-*LacZ* vector carries the *Escherichia coli LacZ* gene (rAd-*LacZ*) described previously (Kozarsky et al., 1993). To confirm the expression of β-galactosidase (LacZ) in the liver, frozen tissue sections (10 μm) were fixed in 0.5% glutaraldehyde (Sigma-Aldrich) at room temperature for 10 min, washed with PBS and stained for 2 h at 37°C in PBS containing 5 mM K3 Fe(CN)_6_, 5 mM K4 Fe(CN)_6_, 1 mM MgCl_2_ (all from Sigma-Aldrich) and 1 mg/ml 5-bromo-4-chloro-3-indolyl-β-D-galactopyranoside (X-gal; Boehringer Mannheim). The sections were then counterstained with Hematoxylin (Thermo Fisher Scientific) and mounted for microscopic evaluation.

### Adenoviral injections

Young (3- to 4-week-old) HIV-Tg_26_ FVB/N mice and their WT littermates were injected through the retro-orbital plexus with 5×10^8^ plaque-forming units (pfu)/mouse of the corresponding rAd-FGF-2 or rAd-*LacZ* vectors using a 0.5 ml insulin syringe (Becton Dickinson). All mice had free access to water and standard food, and were treated in accordance with the National Institutes of Health guidelines for the care and use of research animals. Mice were euthanized at different time points after adenoviral injections.

### Treatment of HIV-Tg_26_ mice injected with rAd-FGF-2 vectors with the FGF/VEGF receptor tyrosine kinase inhibitor PD173074

HIV-Tg_26_ males were divided into two groups (four to five mice per group) and injected via the retro-orbital plexus with 5×10^10^ pfu/mouse of rAd-FGF-2 vectors. Five mice were injected i.p. daily with (1 mg/kg body weight) of the FGF/VEGF tyrosine kinase inhibitor PD173074 from Calbiochem, purchased from Sigma-Aldrich (341607). PD173074 was diluted in 10% dimethyl sulfoxide (DMSO) and injected i.p. daily beginning 3 days after rAd-FGF-2 injection. Control mice (*n*=4 mice) were injected with 10% DMSO diluted in PBS. Mice were euthanized 7 days after the rAd-FGF-2 injection. Serum, urine and kidney samples were collected and stored frozen at −70°C or fixed in formalin, or processed to quantify the proteinuria/albuminuria. Five microliters of urine collected from each mouse were separated by 10% SDS-PAGE and stained with Coomassie Blue.

### Renal injury scores

Kidney sections were fixed in 4% paraformaldehyde and embedded in paraffin, and 5 µm sections were cut and stained with Hematoxylin and Eosin or Masson's Trichrome to highlight the connective tissue. Each kidney cross-section was evaluated blindly using a microscope with 20× and 40× magnification lenses. A total of 50 glomeruli and tubular sections were assessed per group. The following parameters were used to develop a renal injury score: (1) the percentage of glomeruli exhibiting collapse, segmental or global sclerosis; (2) the percentage of enlarged glomeruli, determined by measuring the glomerular area in ten randomly selected juxtamedullary glomeruli per kidney section under a microscope with a 20× amplification lens connected to an Adobe Photoshop program that measures surface areas in kidney sections; these results were expressed as a percentage of the mean values generated in control WT mice infected with rAd-*LacZ* vectors; (3) the percentage of dilated tubules, casts and/or microcysts; and (4) the percentage of interstitial inflammation documented in five randomly selected fields per section examined under a 40× amplification lens, and ranked as 0, no inflammation; 1, minimal inflammation, 2, marked inflammation. The mean values of all these results were added to generate the following renal injury scores: 0, 0-5%; 1, >5-10%; 2, >10-25%; 3, >25-50%; 4, >50%.

### Blood, urine and kidney sample collection

Urine, blood and kidney samples were harvested at different time points as described above, and kept frozen at −80°C. BUN was assessed using a QuantiChrom Urea Assay kit (BioAssay Systems, DIUR-500) as described previously (Mattison et al., 2012). The urinary creatinine levels were measured using a colorimetric assay (R&D Systems, KGE005). Albuminuria was measured with a mouse albumin ELISA kit (Bethyl Laboratories, E99-134) and expressed as a ratio of the urinary creatinine. In addition, SDS-PAGE (4-12%) was carried out with 5 ml urine that was then stained with Coomassie Blue stain solution (Bio-Rad) to detect changes in high- and low-molecular mass urinary proteins as described previously (Das et al., 2016). For measuring the FGF-2 levels, blood was collected in tubes with EDTA, centrifuged at 4000 ***g*** for 5 min and stored immediately at −70°C. FGF-2 secreted by cells transduced with the rAd-FGF-2 vectors was measured with a human FGF basic/FGF-2/Quantikine ELISA Kit (R&D Systems, DFB50), as previously described.

### RT-PCR analysis

Total kidney RNA was isolated using TRIzol (Invitrogen, 15596-026) and treated with deoxyribonuclease I, following Invitrogen's protocol for RT-PCR studies. cDNA was generated from 3 μg RNA using the SuperScript III First-Strand Synthesis System for RT-PCR (Invitrogen, 18080-051). To determine the relative expression of HIV-1 envelope (*env*), we used the following primers: forward primer 5′-TGTGTAAAATTAACCCCACTCTG-3′ and reverse primer 5′-ACAACTTATCAACCTATAGCTGGT-3′. To determine whether the liver and kidney were transduced by the rAd-FGF-2 vectors we used the following human *FGF2* primers: forward primer 5′-CATGGCAGCCGGGAGCATCACC-3′ and reverse primer 5′-TCAGCTCTTAGCAGACATTGG-3. As a control, we amplified the mouse housekeeping gene glyceraldehyde-3-phosphate dehydrogenase (*Gapdh*) using the forward primer 5′-CTTACTCCTTGGAGGCCATGT-3′ and the reverse primer 5′-GCCAAGGTCATCCATGACAAC-3′. During the amplification process, samples were kept at 94°C for 4 min, followed by 35 cycles at 94°C for 30 s, 55°C for 30 s and 72°C for 1 min, and a final extension of 8 min. For each HIV *env*, *FGF2* and *Gapdh* PCR amplification reaction, we used 5 μl and 2 μl cDNA, respectively. The densitometry analysis was conducted using Adobe Photoshop 6.0, as described previously (Li et al., 2017; Xie et al., 2014).

### Real-time RT-PCR analysis

Real-time RT-PCR studies were performed on cDNA samples using a Platinum qPCR SuperMix-UDG kit (Invitrogen, 11730-017). The HIV envelope assay was designed to amplify a 95 bp amplicon from HIV-1 NL4-3 (GenBank accession number AF324493) [forward primer 5′-CCTTTGAGCCAATTCCCATACATT-3′, reverse primer 5′-gacgttTGGTCCTGTTCCATTGAACGTC-3′ with fluorescein amidite (FAM)-labeled LUX]. The mouse *Gapdh* housekeeping gene quantitative PCR control assay was designed to amplify a 93 bp amplicon from (GenBank accession number NM_008084.1) (forward primer 5′-gacatacAGGCCGGTGCTGAGTATGT-3′ with JOE-labeled LUX, reverse primer 5′-TTTGGCTCCACCCTTCAAGT-3′). The real-time PCR amplification protocol was as follows: 50°C for 2 min hold (uracil-DNA glycosylase treatment); 95°C for 2 min; and 40 cycles of 95°C for 15 s, 58°C for 30 s and 72°C for 30 s, using a 7900 Fast Real-Time PCR System (Applied Biosystems). Data were normalized to *Gapdh* and presented as fold increase compared to the rAd-*LacZ* control group. PCR products were resolved on 3% agarose gels.

### Immunohistochemistry

Paraffin-embedded sections (4 μm) were de-paraffinized, rehydrated and stained as described previously (Jerebtsova et al., 2007). Immunostaining was performed with a commercial streptavidin-biotin-peroxidase complex (Histostain SP Kit, Zymed), according to the manufacturer's instructions. The peroxidase activity was monitored after the addition of substrate using a DAB kit (Vector Laboratories, SK-4100) or AEC substrate kit (Invitrogen, 002007). Sections were counterstained with Hematoxylin. FGF-2 was detected in renal tissues as previously described (Ray et al., 1999). Briefly, the renal sections were incubated for 1 h at room temperature with affinity purified IgG fractions (2.5 μg/ml) of a rabbit polyclonal antibody (Ab 773) raised against a unique peptide sequence (1-24) of FGF-2 (provided by Dr Baird, PRIZM Pharmaceutical, San Diego, CA, USA). This antibody was validated in previous rodent and human studies (Gonzalez et al., 1990). To facilitate penetration of the FGF-2 antibody, sections were treated with 1 mg/ml hyaluronidase (type V; Sigma-Aldrich) buffered at pH 5.5 with 0.1 M sodium acetate containing 0.15 M NaC1, for 30 min at 37°C. Nonimmune rabbit IgG was used as a negative control. PCNA was detected using a PCNA staining kit from Invitrogen (931143). pERK was detected with a polyclonal rabbit phospho-pERK (Thr980) antibody (Cell Signaling Technology, 3191; 1:75 dilution). WT-1 staining was assessed with a mouse monoclonal anti-human WT1 antibody (clone 6F-H2, Millipore Sigma; 1:600 dilution). Synaptopodin was detected with a ready-to-use mouse monoclonal antibody (clone G1D4, batch number 1372) from Fitzgerald Industries International (10R-2373). Vimentin was detected with a mouse anti-vimentin antibody (clone LN-6, Sigma-Aldrich; 1:200 dilution). α-SMA was detected with a mouse monoclonal antibody (clone 1A4, Sigma-Aldrich; 1:200 dilution). Macrophages were detected with a rat anti-mouse F4/80 antibody (Bio-Rad, formerly Serotec AbD; 1:20 dilution). Controls included replacing the primary antibody with equivalent concentrations of the corresponding nonspecific antibodies and/or omitting the first or second antibodies. When mouse antibodies were used on mouse sections, the M.O.M. (Mouse on Mouse) ImmPRESS Horseradish Peroxidase (HRP) Polymer Kit (Vector Laboratories, MP-2400) was used. For primary rabbit antibodies, the ImmPRESS HRP Horse Anti-Rabbit IgG (Peroxidase) Polymer Detection Kit (Vector Laboratories, MP-7401) was used. Heat-induced epitope retrieval method was used for PCNA, vimentin, pERK and F4/80 staining. Double immunostaining for PCNA and WT1 in mouse tissues was performed with the M.O.M. ImmPRESS HRP (Peroxidase) Polymer Kit. Three renal sections from young children (<12 years of age) with HIVAN and three controls were obtained from archived autopsies performed at the Children's National Hospital, and stained for PCNA^+^ (clone PC10, Dako), vimentin^+^ (clone V9, Dako), α-SMA^+^ (clone 1A4, Sigma-Aldrich) and CD68^+^ (clone KP1, Dako) macrophages. The latter studies were approved by the institutional review board of the Children's National Hospital with a waiver of consent.

### Western blot analysis

The kidneys were lysed using RIPA lysis buffer containing protease inhibitors and phosphatase inhibitor cocktail 2 (Sigma-Aldrich), and processed by western blotting as described previously (Mattison et al., 2012). The following primary antibodies were used: anti-phospo-p44/42 mitogen-activated protein kinase (Thr202/Tyr204), anti-p44/42 mitogen-activated protein kinase ERK1/2 (both obtained from Cell Signaling Technology, 9101 and 9102, respectively), rabbit polyclonal anti-PCNA (C-20) and goat polyclonal anti-β-actin (I-19) (Santa Cruz Biotechnology, sc-9857 and sc-1616, respectively). All primary antibodies were diluted 1:1000 and incubated overnight at 4°C. Protein bands were detected using Supersignal West Pico Chemiluminescent Substrate (Thermo Fisher Scientific) according to the manufacturer's instructions. All membranes were exposed to Kodak film (X-OMAT) and developed using an automated developer. Densitometry analysis of the data expressed as a β-actin ratio was performed using Adobe Photoshop 6.0, as described previously (Xie et al., 2014).

### Statistical analysis

If not specified otherwise, the data were expressed as mean±s.e.m. Differences between two groups were compared using Mann–Whitney unpaired *t-*test. Multiple sets of data were compared by one-way ANOVA with Newman–Keuls post-hoc comparisons. Statistical analyses were performed using Prism software (version 5.00; GraphPad Software). Values of *P*<0.05 were considered statistically significant.

## Supplementary Material

Supplementary information
