# Supplementary material for: Circulating fibroblast growth factor-2 precipitates HIV nephropathy in mice
Source: Dis Model Mech. 2021 Jul 26;14(7):dmm048980. doi: 10.1242/dmm.048980 (PMC8326767; doi:10.1242/dmm.048980)
Supplement: Supplementary information [file dmm-14-048980-s1.pdf]

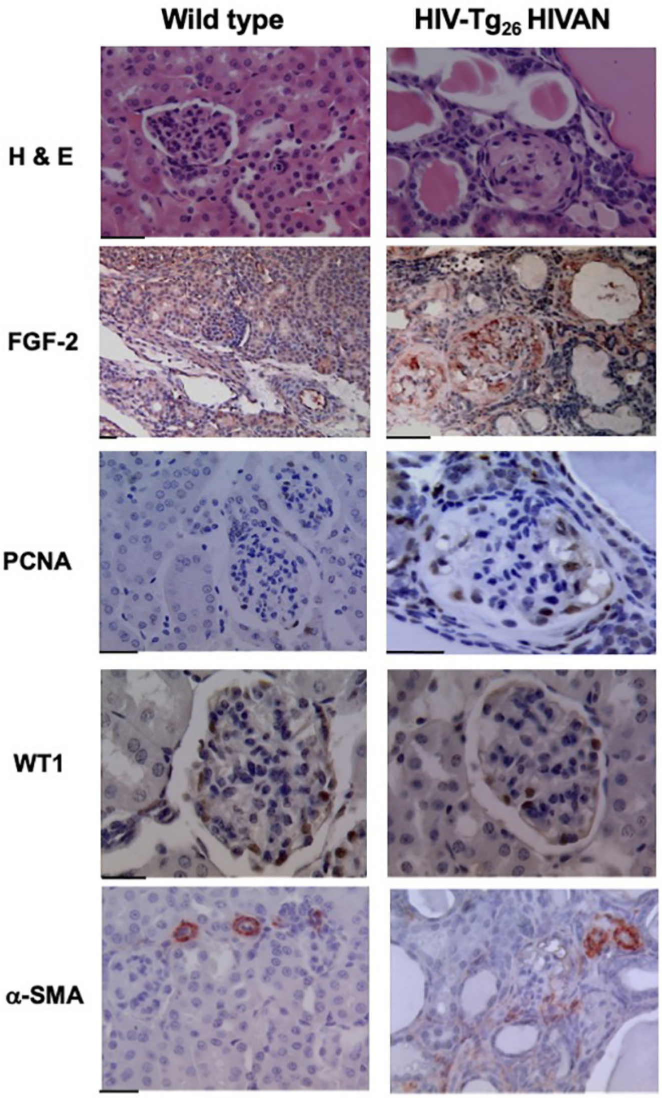

Fig S1. HIVAN associated nephropathy (HIVAN) in HIV-Tg$_{26}$ mice. The panels show representative renal sections from wild type and HIV-Tg$_{26}$ mice which developed HIVAN spontaneously. Renal sections were stained with hematoxylin and eosin (H&E), or processed for immunohistochemistry studies to detect Fibroblast Growth Factor-2 (FGF-2), Proliferating Cell Nuclear Antigen (PCNA), Wilms Tumor 1 antigen (WT1), and α-smooth muscle actin (α-SMA) as described in Material and Methods. Renal sections from HIV-Tg$_{26}$ mice with HIVAN showed glomerular sclerotic lesions with microcystic tubular dilatation and casts, recruitment of FGF-2 in

Disease Models & Mechanisms • Supplementary information

renal glomeruli and tubulo-interstitium (red color), several glomerular and tubular epithelial PCNA+ cells (brown color), few WT1+ cells in renal glomeruli (brown color), and recruitment of α-SMA + cells in the tubulo-interstitium (red color). Renal vessels from wild type and HIV-Tg_{26} mice also stained positive for α-SMA. Scale bars: 20 μm. (n = 5 mice per group).
